# Triglyceride to high-density lipoprotein cholesterol ratio associated with long-term adverse clinical outcomes in patients deferred revascularization following fractional flow reserve

**DOI:** 10.1186/s12944-024-02093-1

**Published:** 2024-04-02

**Authors:** Fanqi Li, Xiaofang Li, Jiabao Zhou, Qiuzhen Lin, Yong Zhou, Jiayi Zhu, Keke Wu, Donghui Zhao, Qiuyu Li, Haixiong Wang, Qiming Liu

**Affiliations:** 1 Department of Cardiology, The Second Xiangya Hospital of Central South University, Changsha, 410000 China; 2 Department of Digestive Oncology, Tongji Shanxi Hospital, Shanxi Bethune Hospital, Shanxi Academy of Medical Sciences, Third Hospital of Shanxi Medical University, Taiyuan, Shanxi 030032 China; 3 Department of Cardiology, Beijing Anzhen Hospital, Capital Medical University, Beijing, 100029 China; 4 Department of Cardiology, Shanxi Cardiovascular Hospital, Taiyuan, Shanxi 030001 China

**Keywords:** TG/HDL-C, Fractional flow reserve, Coronary artery disease, Prognosis

## Abstract

**Background:**

Guidelines on coronary intermediate lesions strongly recommend deferred revascularization after detecting a normal fractional flow reserve (FFR). Researches about triglyceride to high-density lipoprotein cholesterol (TG/HDL-C) on cardiovascular diseases has also been well conducted. However, the association of TG/HDL-C and long-term adverse clinical outcomes remains unknown for patients deferred revascularization following FFR.

**Methods:**

This study retrospectively included 374 coronary artery disease (CAD) patients with non-significant coronary lesions diagnosed by coronary angiography (CAG) and FFR. The main outcome measure was the combination of major adverse cardiovascular and cerebrovascular events (MACCEs). All patients were categorized into three subgroups in terms of TG/HDL-C tertiles (T1 < 0.96, 0.96 ≤ T2 < 1.58, T3 ≥ 1.58). Three different Cox regression models were utilized to reveal the association between TG/HDL-C and prevalence of MACCEs.

**Results:**

47 MACCEs were recorded throughout a median monitoring period of 6.6 years. The Kaplan-Meier survival curves showed a higher MACCEs rate occurred in the higher TG/HDL-C group (5.6% vs. 12.9% vs. 19.4%, log-rank *P* < 0.01). After adjustment, patients in T3 suffered a 2.6-fold risk compared to the T1 group (T3 vs. T1: HR 2.55, 95% CI 1.05–6.21, *P* = 0.038; T2 vs. T1: HR 1.71, 95% CI 0.65–4.49, *P* = 0.075; *P* for trend = 0.001). The restricted cubic spline (RCS) analysis demonstrated that the HR for MACCEs rose as TG/HDL-C increased. Both the receiver operating characteristic (ROC) and time-dependent ROC proved the excellent predictive ability of TG/HDL-C.

**Conclusion:**

The study illustrates that TG/HDL-C correlates with the risk of MACCEs in CAD patients deferred revascularization following FFR. TG/HDL-C could serve as a dependable predictor of cardiovascular events over the long term in this population.

**Supplementary Information:**

The online version contains supplementary material available at 10.1186/s12944-024-02093-1.

## Introduction

Determining whether revascularization for coronary intermediate stenosis presents a significant challenge for cardiovascular interventionalists, defined as having a 40–70% obstruction as visually estimated [1]. FFR, which is calculated based on the mean distal stenosis pressure and aortic pressure, is strongly advocated for assessing these intermediate lesions for its physiologic function detection ability [1, 2]. FFR > 0.8 indicates that the lesion is non-significant coronary stenosis and deferred revascularization is recommended. With evidence accumulation, other determinants on prognosis emerged, including the plaque vulnerability and progression [3], the interference of microvascular dysfunction on FFR [4], and other non-coronary factors. A recent meta-analysis containing 4275 patients deferred revascularization following a negative FFR concluded that the cardiovascular events of diabetic patients was 2.08 folds greater than non-diabetic [5]. Hence, it is crucial to screen high-risk populations for patients deferred revascularization and administer more comprehensive treatment, even if with a normal FFR, in order to decrease the occurrence of MACCEs.

Dyslipidemia, identified as a conventional risk factor for CAD, includes a range of lipid particle disorders, such as low-density lipoprotein cholesterol (LDL-C), HDL-C, total cholesterol, and TG, with the recent addition of lipoprotein (a) to this list. LDL-C has been the subject of extensive researches and lowering LDL-C level has been confirmed an effective therapeutic option by clinical practice. Recently, enormous studies have proved the relationship between components of lipids and CAD, besides LDL-C. Among these factors, TG/HDL-C is closely linked to cardiovascular metabolic diseases [6]. Nonetheless, the effect of this ratio still unknown among patients deferred revascularization following FFR. Thus, the study intended to explore association of TG/HDL-C with long-term cardiovascular events in this group.

## Methods

### Study population

**Fig. 1:**
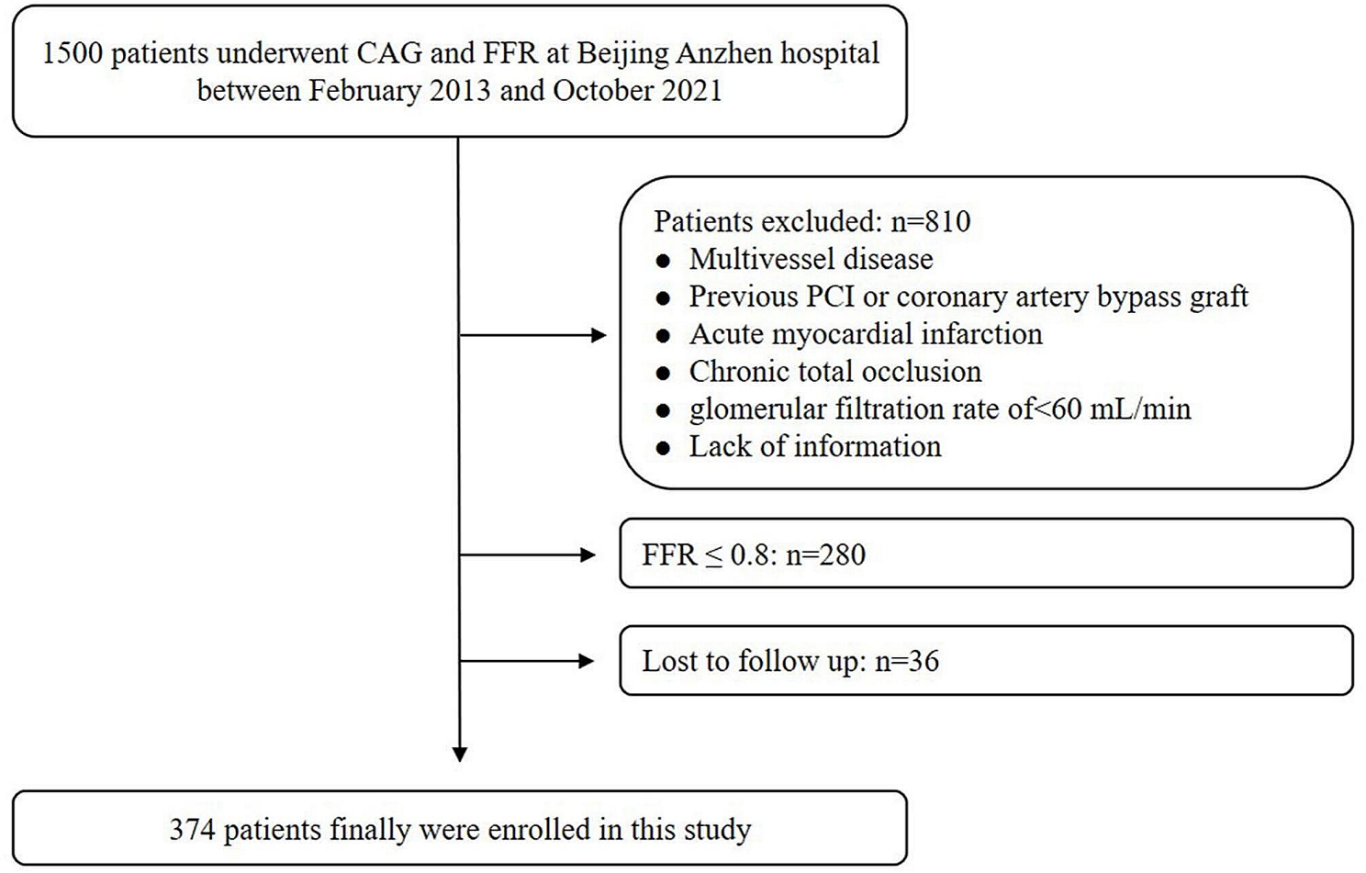
Population flow chart of enrolled patients. CAG, coronary angiography; FFR, fractional flow reserve; PCI, percutaneous coronary intervention

This study retrospectively included 1500 consecutive participants who received FFR and CAG for coronary intermediate lesions from February 2013 to October 2021, the same as the previous study population [7]. The study design was approved and performed in Beijing Anzhen Hospital, which is a major referral center dedicated to cardiovascular disease. The exclusion criteria were also similar to our previous work and are illustrated in Fig. 1. The main difference in this study is that only FFR > 0.8 out of the 1500 consecutive patients were included for analysis. Ultimately, 374 patients participated in this study. This study design was approved by The Second Xiangya Hospital of Central South University and Beijing Anzhen Hospital.

### Interventional procedures

FFR and CAG procedures were performed by skilled operators in strict compliance with protocols. Should the CAG reveal intermediate stenosis, as determined by at least two seasoned interventionalists, an FFR evaluation is scheduled to be proceed, contingent upon patient and family consent. FFR findings informed decisions about whether PCI. Patients with a positive FFR results that indicated PCI but family members refused were excluded. The selection of specific stent size and perioperative drugs was at the discretion of the physician. No major complications occurred during interventional operation in all patients.

### Baseline information collection and definition

Baseline information was acquired from electronic medical records system after approval by the ethics review committee. On the morning after admission, a fasting venous blood sample was collected then examined in Department of Laboratory Medicine. Monitoring information was gathered via electronic medical records system and through phone calls until October 2023. Diabetes mellitus and hypertension were diagnosed following globally recognized standards. Smoking status was identified if the individual had smoked within the six months leading up to the follow-up. FFR represents the ratio of the average pressure in distal stenosis to the aortic pressure at maximal myocardial perfusion.

### Primary endpoints

The median follow-up period in this study up to 79 months. The primary outcome measured was the composite of MACCEs, encompassing nonfatal stroke, repeat revascularization, all-cause death, and nonfatal myocardial infarction. Nonfatal stroke was characterized by the rapid onset of clinical symptoms indicating cerebral dysfunction persisting for over 24 h, supported by neuroimaging evidence [8]. The other three diagnoses were also made according to official guidelines. All MACCEs occurrence events were recorded and compared in the three groups stratified by TG/HDL-C tertiles.

### Statistical analysis

Continuous variables were commonly showed as either the mean ± SD or the median (P25, P75), on the basis of their distribution normality. One-way ANOVA was utilized for comparing normally distributed data, while the Kruskal-Wallis test was calculated for non-normally distributed data. Categorical variables were depicted as counts and percentages, with chi-square or Fisher’s exact test employed for statistical analysis. Patients were stratified into three subgroups according to TG/HDL-C tertiles. The incidence of MACCEs was recorded using Kaplan-Meier curves, and differences among the sets were calculated with the log-rank test. Three different Cox regression models were constructed to explore the relationship between graded TG/HDL-C tertiles and MACCEs risk. Model 1 adjusted for sex and age; Model 2 adjusted for sex, age, diabetes mellitus, BMI, smoking, and hypertension; Model 3 adjusted for sex, age, diabetes mellitus, BMI, smoking, hypertension, EF, CRP, LDL-C, uric acid, and FFR. An RCS analysis using 4 knots was plotted to explore relationship between TG/HDL-C (as continuous variable) and MACCEs risk. TG/HDL-C and FFR were incorporated in logistic regression to generate a new “predict” variable, which was intended to plot receiver operating characteristic (ROC) curve. ROC curves were utilized to compare the predictive capacity of different markers, and time-dependent ROC analysis examined the predictive power of TG/HDL-C at different time points. Differences in AUCs compared to TG/HDL-C were assessed using the Delong test. Data analysis was performed with SPSS 26.0, and visualization was done with GraphPad Prism 9.5.0 and R language software (R 4.1.3). For all calculations, a significance level of *P* < 0.05 was employed.

## Results

Ultimately, this study enrolled a total of 374 patients, and Fig. 1 displays the selection process flow chart. The average age was 58.63 ± 9.05 years, and 252 (67.4%) individuals were men. The mean value of TG/HDL-C was 1.49 ± 1.04, and tertiles were T1 < 0.96, 0.96 ≤ T2 < 1.58, T3 ≥ 1.58. Throughout a median follow-up duration up to 79 months, 47 cases of MACCEs were documented, accounting for 12.6%.

### Baseline characteristics

The baseline information stratified by the TG/HDL-C tertiles were exhibited in Table 1. Patients in T3 group are more likely to have dyslipidemia, higher BMI, LDL-C, TG, TC, TG/HDL-C, Glu, UA, CRP, and lower HDL-C compared to the low TG/HDL-C group (*P* < 0.05).

**Table 1:** Baseline characteristics of patients

	TG/HDL-C level			P
	T1(< 0.96)	T2(≥ 0.96, < 1.58)	T3(≥ 1.58)	
N	126	124	124	
Age(y)	59.46 ± 9.39	58.69 ± 9.07	57.73 ± 8.67	0.320
Male, n (%)	81 (64.29)	78 (62.90)	93 (75.00)	0.084
BMI, kg/m^2^	24.76 ± 2.70	25.83 ± 2.49	25.85 ± 2.71	0.001
Risk factors, n (%)				
Smoking	31 (24.60)	35 (28.23)	45 (36.29)	0.118
Hypertension	64 (50.79)	74 (59.68)	70 (56.45)	0.359
Diabetes mellitus	31 (24.60)	32 (25.81)	35 (28.23)	0.803
Dyslipidemia	59 (46.83)	67 (54.03)	78 (62.90)	0.038
Laboratory results				
RBC, 10^12^/L	4.55 ± 0.36	4.59 ± 0.49	4.67 ± 0.45	0.102
WBC, 10^12^/L	5.89 (5.00,6.96)	6.25 (5.30,7.51)	6.38 (5.34,7.47)	0.185
PLT, 10^9^/L	222.00 (184.00,254.00)	223.00 (186.75,250.25)	212.50 (181.00,244.00)	0.601
LDL-C, mmol/L	2.11 (1.67,2.72)	2.42 (1.76,2.83)	2.59 (2.03,3.12)	< 0.001
HDL-C, mmol/L	1.35 (1.16,1.55)	1.10 (0.98,1.23)	0.94 (0.85,1.05)	< 0.001
TC, mmol/L	3.84 (3.31,4.63)	3.95 (3.30,4.63)	4.35 (3.73,5.09)	< 0.001
TG, mmol/L	0.86 (0.67,1.00)	1.37 (1.16,1.56)	2.21 (1.85,2.75)	< 0.001
Glu, mmol/L	5.50 (5.04,6.44)	5.61 (5.24,6.52)	5.83 (5.39,6.86)	0.010
HbA1c, %	5.80 (5.40,6.30)	5.90 (5.57,6.62)	5.95 (5.60,6.82)	0.076
ALT, mmol/L	21.00 (14.00,27.00)	21.50 (15.00,30.00)	22.00 (16.00,33.00)	0.139
AST, mmol/L	20.00 (18.00,24.00)	21.50 (19.00,26.00)	21.00 (17.75,25.25)	0.126
TP, g/L	69.32 ± 5.74	68.98 ± 5.29	69.51 ± 5.65	0.753
Urea, mmol/L	5.09 (4.50,5.80)	5.10 (4.40,6.20)	5.40 (4.35,6.10)	0.703
UA, µmol/L	310.05 (271.35,338.32)	315.55 (292.32,346.33)	324.45 (299.15,361.45)	0.012
Cr, µmol/L	65.55 (56.00,72.68)	68.00 (58.90,74.85)	69.10 (60.93,78.03)	0.017
Hcy, µmol/L	11.25 (8.72,14.57)	11.90 (9.38,14.45)	12.00 (9.10,16.30)	0.409
CRP, mg/L	0.76 (0.40,1.54)	0.91 (0.37,2.04)	1.10 (0.69,2.42)	0.010
EF, %	65 (61,67)	65 (62,68)	65 (62,68)	0.485
TG/HDL-C	0.65 (0.49,0.80)	1.25 (1.11,1.38)	2.25 (1.91,2.75)	< 0.001
Procedure characteristics				
FFR	0.85 (0.83,0.88)	0.85 (0.82,0.88)	0.85 (0.83,0.88)	0.729
LM, n (%)	3 (2.4)	2 (1.6)	2 (1.6)	0.654
LAD, n (%)	90 (71.4)	90 (72.6)	82 (66.1)	0.496
LCX, n (%)	10 (7.9)	12 (9.7)	15 (12.1)	0.543
RCA	23 (18.3)	20 (16.1)	25 (20.2)	0.712
Medication at discharge, n (%)				
Aspirin	124 (98.4)	122 (98.4)	124 (100)	0.367
Statin	93 (73.8)	96 (77.4)	104 (83.9)	0.148
Ezetimibe	17 (13.5)	21 (16.9)	26 (21.0)	0.291
TG-lowering agents	2 (1.6)	4 (3.2)	18% (6.5)	0.120
β-Blocker	54 (42.9)	69 (55.6)	60 (48.4)	0.128
antidiabetic agents	22 (17.5)	27 (21.8)	32 (25.8)	0.277

TG/HDL-C, Triglyceride/High-density lipoprotein cholesterol; BMI, body mass index; RBC, red blood cell; WBC, white blood cell; PLT, platelet; LDL-C, low-density lipoprotein cholesterol; HDL-C, high-density lipoprotein cholesterol; TC, total cholesterol; TG, triglyceride; Glu, glucose; HbA1c, glycosylated hemoglobin; ALT, alanine aminotransferase; AST, aspartate aminotransferase; TP, total protein; UA, uric acid; Cr, creatinine; Hcy, homocysteine; CRP, C reactive protein; EF, ejection fraction; FFR, fractional flow reserve; LM, left main coronary artery; LAD, left anterior descending; LCX, left circumflex; RCA, right coronary artery

### Association between TG/HDL-C and MACCEs

**Fig. 2:**
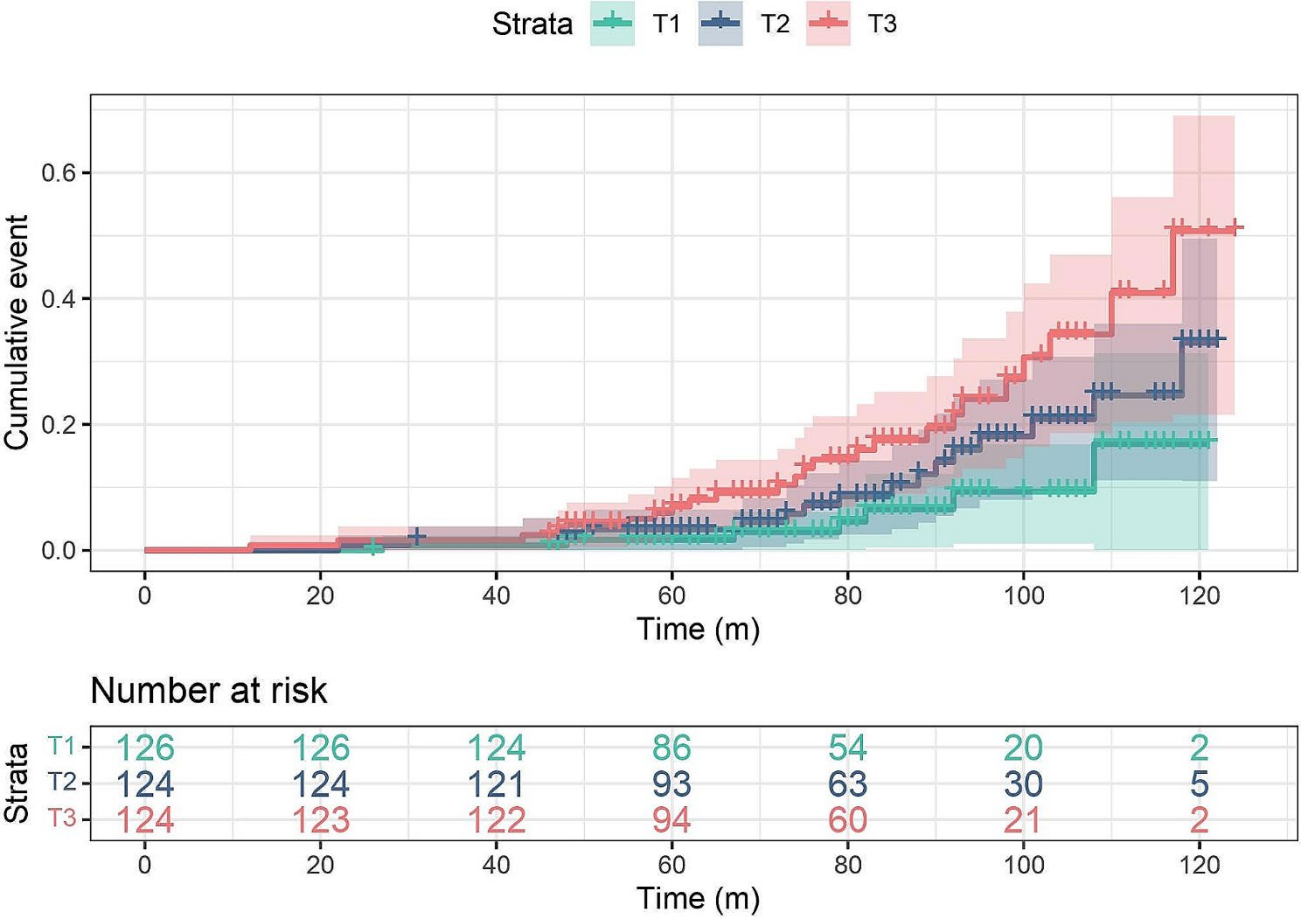
Kaplan-Meier curves for the incidence of MACCEs among the three groups of TG/HDL-C. T1 < 0.96; 0.96 ≤ T2 < 1.58; T3 ≥ 1.58

Kaplan-Meier curves were utilized to depict the cumulative risk of adverse clinical outcomes across the three groups (Fig. 2). Over the duration of the follow-up, there were 7, 16, and 24 MACCEs occurred in the T1, T2, and T3 groups, respectively. As the tertiles of TG/HDL-C increased, significant escalation happened in the cumulative risk of MACCEs (log-rank *P* = 0.0026).

Three different regression models were constructed to analyze the HR of MACCEs for TG/HDL-C. (Table 2). When fully adjusted, the patients in T2 and T3 group encountered a greater risk of MACCEs compared to those in T1 group (Model 3, T2 vs. T1: HR 1.71, 95% CI 0.65–4.49; T3 vs. T1: HR 2.55, 95% CI 1.05–6.21, *P* for trend = 0.001).

**Table 2:** Relationship between TG/HDL-C tertiles and cardiovascular events

	HR (95% CI)					
	Model 1	P	Model 2	P	Model 3	P
T1	1 (reference)		1 (reference)		1 (reference)	
T2	1.96 (0.80–4.78)	0.141	1.32 (0.52–3.37)	0.175	1.71 (0.65–4.49)	0.075
T3	3.55 (1.53–8.24)	0.008	2.58 (1.09–6.11)	0.032	2.55 (1.05–6.21)	0.038

Model 1: adjust for sex and age; Model 2: Model 1 + smoking, hypertension, diabetes mellitus, BMI; Model 3: Model 2 + EF, CRP, LDL-C, uric acid, FFR.

**Fig. 3:**
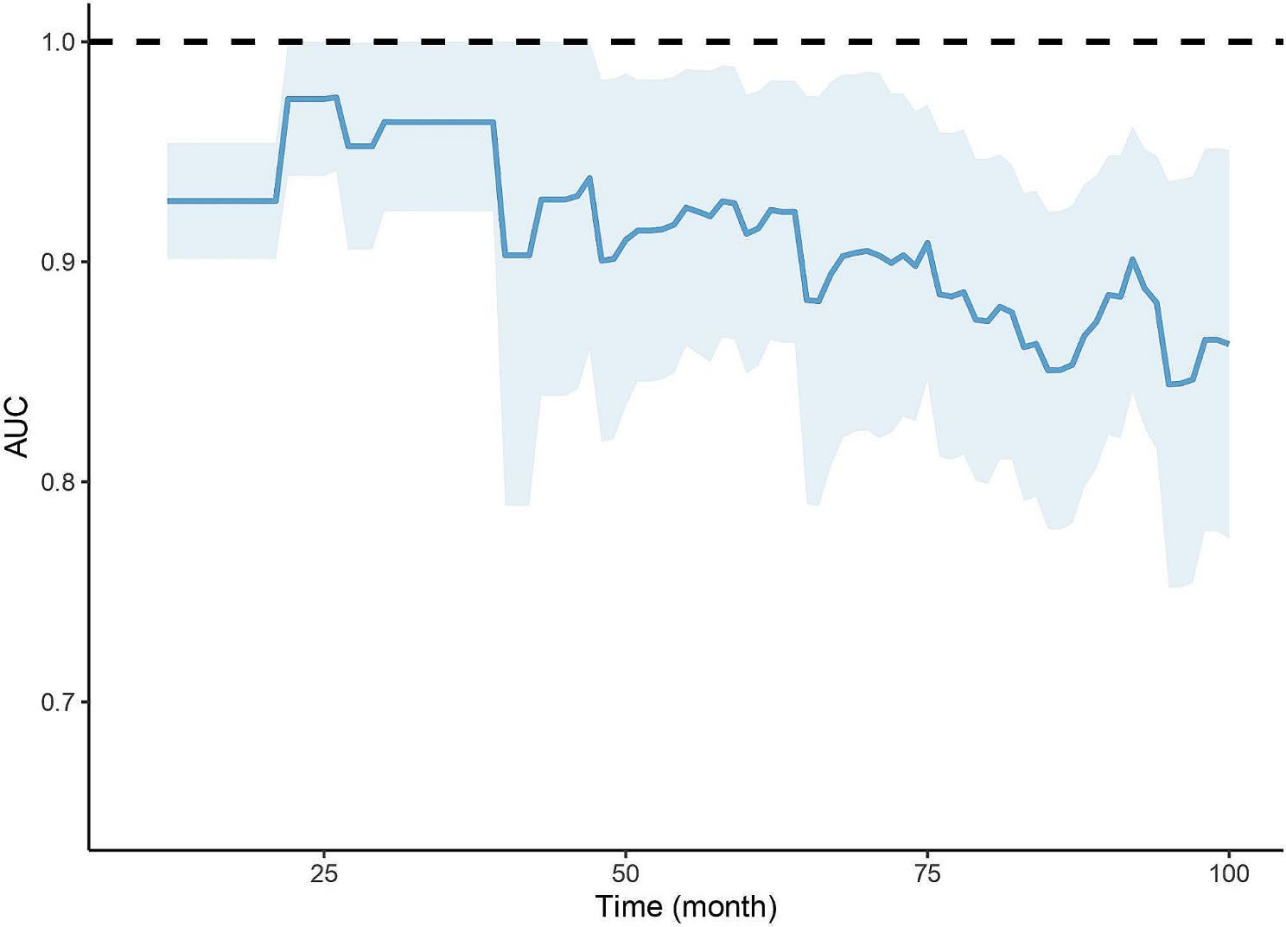
The restricted cubic spline of HR for MACCEs and TG/HDL-C. TG/HDL-C, triglyceride to high-density lipoprotein cholesterol; CI, confidence interval; HR, hazard ratio

To further investigate the possible nonlinear relationship, an RCS curve was conducted (Fig. 3). The likelihood of MACCE events will significantly rise when TG/HDL-C over 1.28, and HR escalated as the ratio TG/HDL-C increasing.

**Fig. 4:**
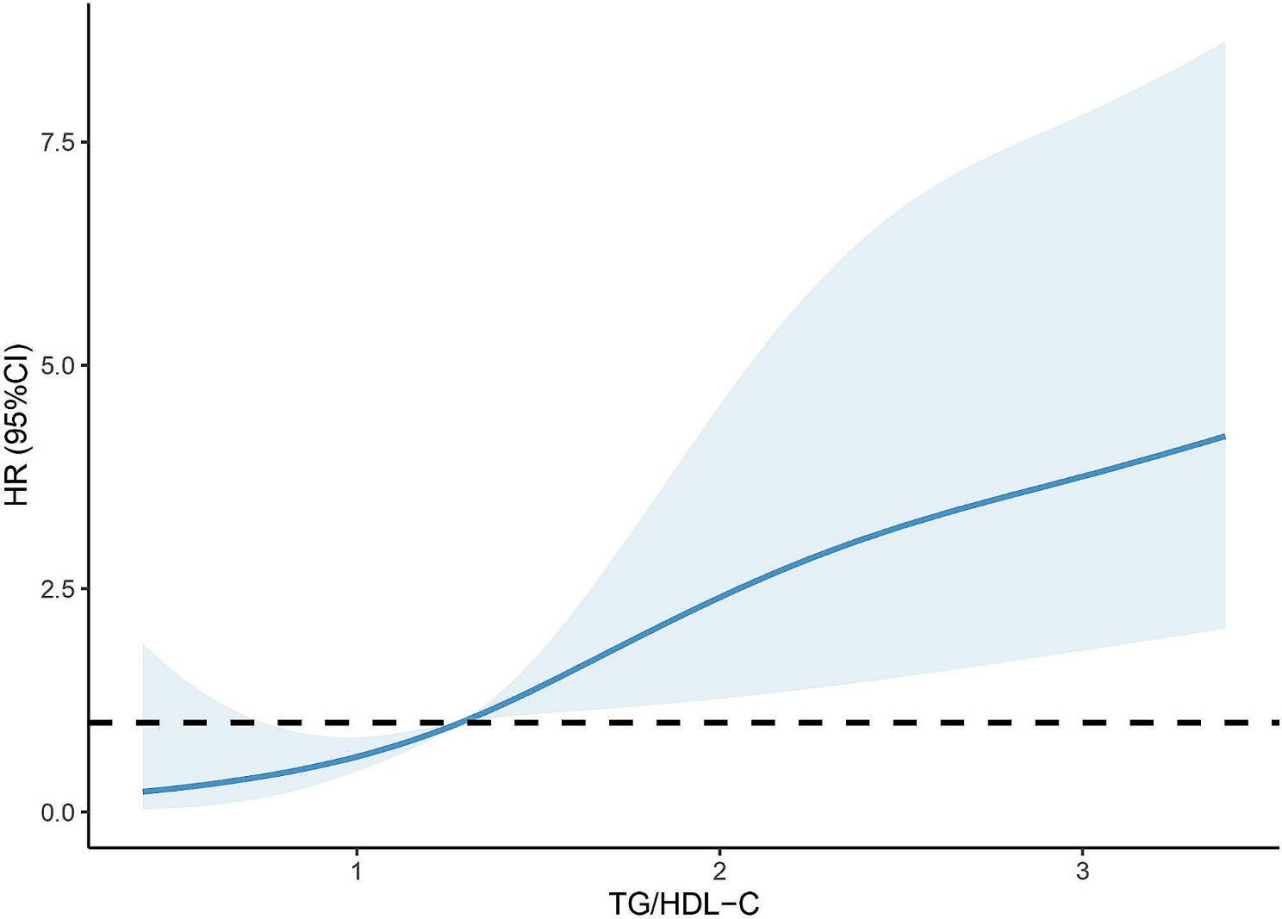
ROC curves of TG/HDL-C and other markers for the incidence of MACCEs. TG/HDL-C, triglyceride to high-density lipoprotein cholesterol; BMI, body mass index; LDL-C, low-density lipoprotein cholesterol; TG, triglyceride; HDL-C, high-density lipoprotein cholesterol

**Fig. 5:**
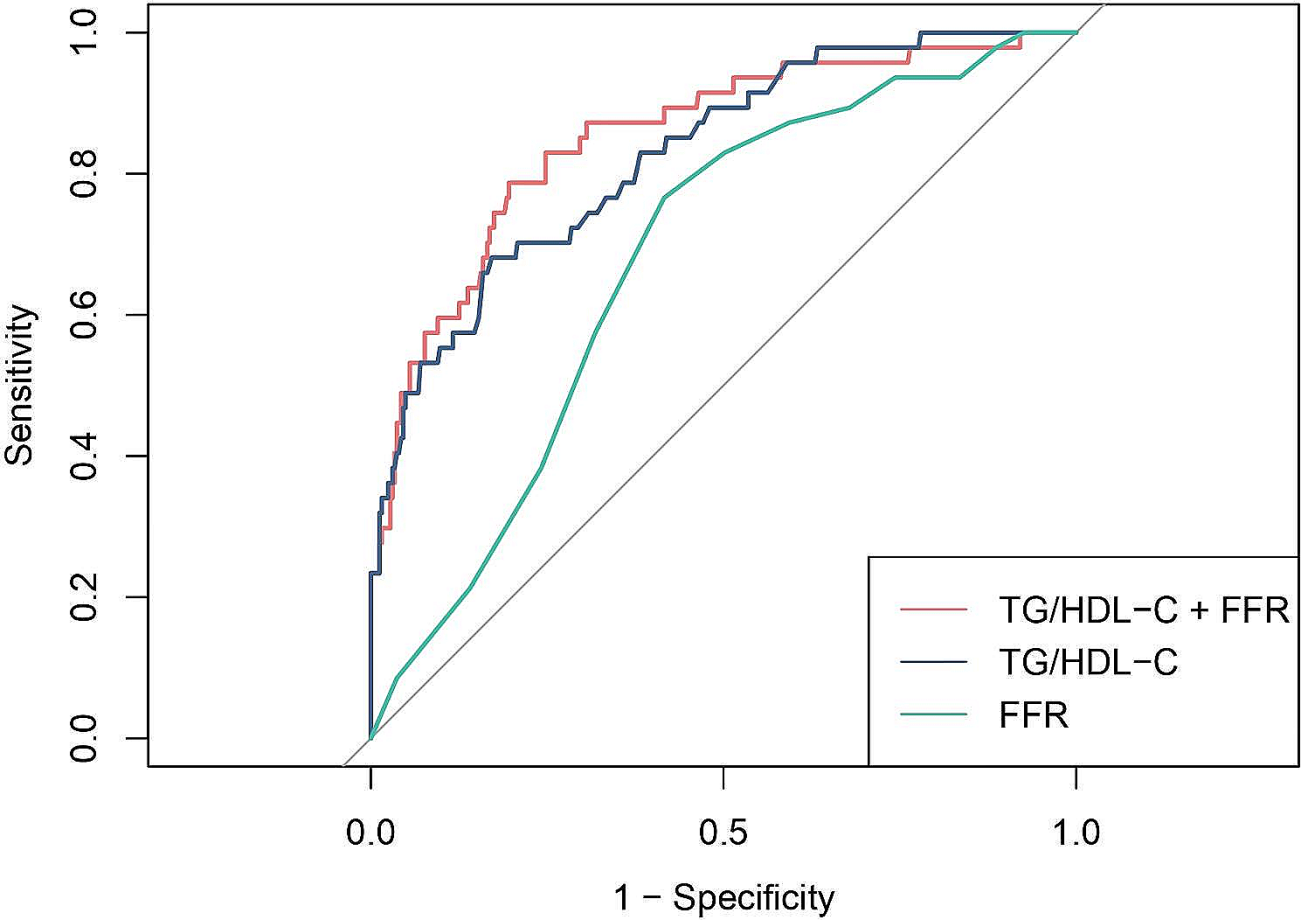
Time-dependent ROC curves of TG/HDL-C for the incidence of MACCEs.

**Fig. 6:**
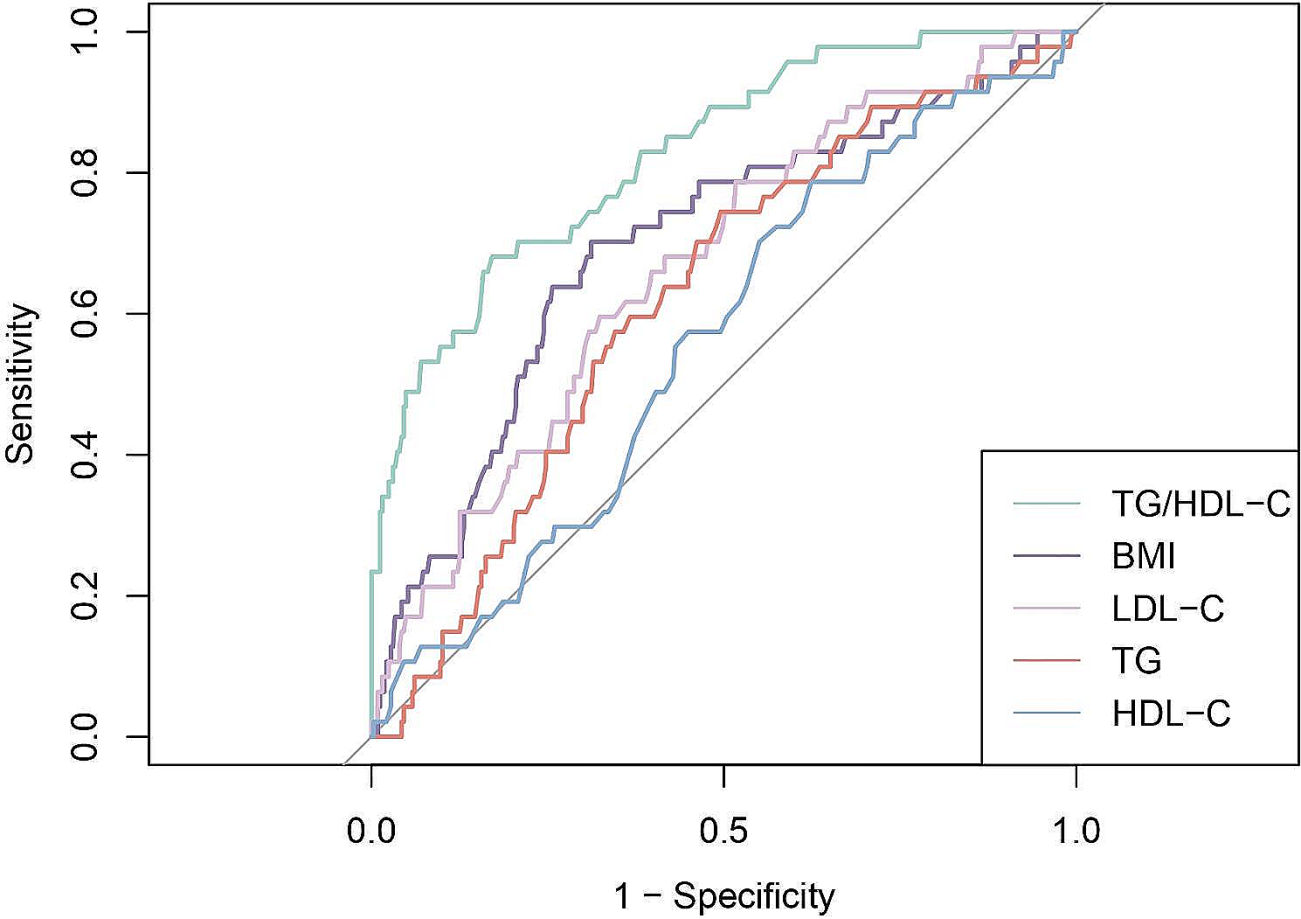
ROC curves of TG/HDL-C, FFR and combination of TG/HDL with FFR for the incidence of MACCEs. TG/HDL-C, triglyceride to high-density lipoprotein cholesterol; FFR, fractional flow reserve

ROC and time-dependent ROC were utilized to detect the predictive capacity of the ratio TG/HDL-C. The findings indicated that TD/HDL-C exhibited a larger AUC compared to BMI, LDL-C, TG, and HDL-C (Fig. 4). The statistical difference of AUCs and the specific AUC values of each indicator were clearly displayed in Table 3. Time-dependent ROC curves also exhibited satisfactory predictive value at different times (Fig. 5). For patients with deferred revascularization, the baseline FFR value also has important predictive value for their long-term prognosis. The combination of TG/HDL-C with FFR exhibited better diagnostic efficacy than either one alone (Fig. 6; Table 3).

**Table 3:** AUC values (95% CI) and difference from TG/HDL-C for each item

	TG/HDL-C	BMI	LDL-C	TG	HDL-C	FFR	TG/HDL-C + FFR
AUC (95%CI)	0.83(0.76–0.89)	0.70(0.62–0.78)	0.67(0.59–0.75)	0.62(0.54–0.70)	0.56(0.48–0.64)	0.68(0.60–0.75)	0.85(0.79–0.91)
Difference	-	0.13	0.16	0.20	0.27	0.15	-0.03
*P*	-	0.02	< 0.01	< 0.01	< 0.01	< 0.01	0.05

TG/HDL-C, Triglyceride/High-density lipoprotein cholesterol; BMI, body mass index; LDL-C, low-density lipoprotein cholesterol; TG, triglyceride; HDL-C, high-density lipoprotein cholesterol; FFR, fractional flow reserve

## Discussion

This study revealed long-term prognostic value of TG/HDL-C in CAD patients who deferred revascularization following a normal FFR assessment for the first time. First, the research findings indicated that patients with elevated TG/HDL-C were at a greater risk of cardiac events than those with lower ratio. Second, the detrimental effects of TG/HDL-C will be significant and aggravated as the ratio increase. Third, TG/HDL-C could act as a dependable predictive factor to predict MACCEs in patients deferred revascularization following FFR.

Revascularization in CAD patients should follow strict criteria, including those on optimal drug treatment still experiencing angina, and those diagnosed with severe stenosis through coronary angiography (CAG). Nevertheless, the decision of whether perform revascularization for patients with intermediate stenosis cloud be ambiguous when based exclusively on CAG. Several strategies are used in current practice to assess the functional significance of coronary lesions and further guide coronary interventional therapy [8]. Among them, FFR has emerged as the “gold standard” in detecting functional ischemia, supported by a wealth of accumulated evidence. FFR-guided PCI has been proved to yield greater benefits than angiography-guided [9, 10]. As evidence continues to grow, authoritative guidelines have classified FFR as IA recommendation for intermediate stenosis [11]. FFR > 0.8 indicates that the lesion is non-significant coronary stenosis and deferred revascularization is recommended. However, it has become evident that the outcomes of deferred revascularization may not be uniform across all patient subgroups, as the utilization of physiology-guided revascularization increases. A recent meta-analysis containing 4275 patients deferred revascularization following negative FFR concluded that the cardiovascular events of diabetic patients was 2.08 folds greater than non-diabetic [5]. A large multinational study demonstrated that sex differences existed in the prognosis among patients with negative FFR and the HR of males for patient-oriented composite outcome (including revascularization, myocardial infarction, and death) was 2.07 [12]. On the other hand, the pathophysiologic mechanisms underlying CAD are also worth considering. First, plaque vulnerability and progression are major contributors to MACCEs, which can’t be well detected by FFR [13]. The COMBINE OCT-FFR trial revealed that thin-cap fibroatheroma positive patients suffered a five-fold higher risk of MACE despite this patients with a negative FFR [14]. Second, the evaluation of coronary stenosis severity using FFR cloud be partially confounded by microvascular dysfunction [15]. More importantly, both atherosclerotic plaque vulnerability and microvascular dysfunction are associated with dyslipidemia [16]. This study findings uncovered the association between TG/HDL-C ratio, two important particles of dyslipidemia, and long-term adverse clinical outcomes among CAD patients who have a normal FFR.

The influence of TG and HDL-C on cardiovascular disease development remains a subject of heated debate, given the unclear effects and contradictory findings from clinical research. Triglycerides are fat molecules formed from 3 molecules of long chain fatty acids and glycerol. HDL-C is a lipoprotein that transports cholesterol from body tissues to the liver. Adequate research confirms the relationship between TG and cardiovascular disease; however, more importantly, there are conflicting conclusions about the ability of this intervention to achieve significant clinical benefit in randomized controlled studies [17]. The fibrates and omega-3 fatty acids, two classic types of TG-lowering drugs, exerted variable clinical results [18–20]. Similarly, the same phenomenon occurred in HDL-C [21]. With the birth of PCSK9 and clinical practice, more and more studies are focusing on patients achieving optimal LDL-C levels and finding that residual cardiovascular risk will be increased if these patients have combined dyslipidemia, particularly for low HDL-C concentrations and high TG levels [22]. However, it is evident that the ratio of TG to HDL-C is linked with cardiovascular events. For example, the widely studied and hot metric: atherogenic index of plasm, is calculated from TG and HDL-C. This study findings similarly suggest that TG/HDL-C possesses superior predictive capacity than TG and HDL alone. Ample studies have been conducted concerning the connection between TG/HDL-C ratio and severity of CAD, covering aspects including metabolic syndrome, insulin resistance, and the existence of high-risk coronary plaques [23–25]. Besides, TG/HDL-C also linked to adverse cardiovascular outcomes for CAD patients [26, 27]. As previously mentioned, as the shortcomings of FFR in identifying unstable plaques and vulnerability to other factors, this close connection of TG/HDL-C and CAD may play a greater role in patients with immediate lesion to assist in screening high-risk patients. Similar to these studies, this research proved that higher level of TG/HDL-C increased risk of MACCEs in patients deferred revascularization following FFR.

FFR related indicators, such as iFR, post-PCI FFR, CT-FFR, are strongly associated with prognosis. The reason why FFR is inferior to TG/HDL-C may be related to the following two points in current study. First, we all know that the cutoff value of FFR is controversial, and even the concept of “gray zone”. Second, this research design only included patients with FFR > 0.8. The range of FFR changed from 0 to 1 to 0.8-1, which may diminish the predict power of FFR. Furthermore, integrating FFR with the TG/HDL-C ratio demonstrated the highest predictive capability, indicating that a predictive model that combines interventional assessment and laboratory results could have a more significant impact on clinical treatments.

## Study strengths and limitations

This study has several strengths. First, it enriches prognosis information of the population with deferred revascularization following FFR, which has rarely been studied. Second, the prognostic value of TG/HDL-C on long-term incidence of MACCEs were investigated in CAD patients who deferred revascularization following a normal FFR assessment for the first time. Third, attention should be paid to the management of the levels of lipid components in patients, not only LDL-C. On the other hand, several limitations also exist. First, the study was conducted at a single-center and was retrospective in nature, which may introduce selection bias or potential confounding variables. Second, TG and HDL-C were only assessed upon admission, with no record of their dynamic changes throughout the follow-up period.

## Conclusion

The study illustrates that TG/HDL-C correlates with the risk of MACCEs in CAD patients deferred revascularization following FFR. TG/HDL-C could serve as a dependable predictor of long term cardiovascular events in this population.

### Electronic supplementary material

Below is the link to the electronic supplementary material.

Supplementary Material 1

Supplementary Material 2

## Data Availability

No datasets were generated or analysed during the current study.
