# Supplementary material for: Triglyceride to high-density lipoprotein cholesterol ratio associated with long-term adverse clinical outcomes in patients deferred revascularization following fractional flow reserve
Source: Lipids Health Dis. 2024 Apr 2;23:96. doi: 10.1186/s12944-024-02093-1 (PMC10985980; doi:10.1186/s12944-024-02093-1)
Supplement: Supplementary file 1 — Supplementary Material 1 [file 12944_2024_2093_MOESM1_ESM.pdf]

20240322204637968654088735453184

WORD COUNT	3986	TIME SUBMITTED	22-MAR-2024 08:47PM
		PAPER ID	107755881

Triglyceride to high-density lipoprotein cholesterol ratio associated with long-term adverse clinical outcomes in patients deferred revascularization following fractional flow reserve

Fanqi Li[1], Xiaofang Li[2], Jiabao Zhou[1], Qiuzhen Lin[1], Yong Zhou[1], Jiayi Zhu[1], Keke Wu[1], Donghui Zhao[3], Qiuyu Li[3], Haixiong Wang, MD & PhD[4*], Qiming Liu, MD & PhD[1*]

1, Department of Cardiology, The Second Xiangya Hospital of Central South University, Changsha 410000, China

2, Department of Digestive Oncology, Shanxi Bethune Hospital, Shanxi Academy of Medical Sciences, Tongji Shanxi Hospital, Third Hospital of Shanxi Medical University, Taiyuan, Shanxi, 030032, China

3, Department of Cardiology, Beijing Anzhen Hospital, Capital Medical University, Beijing 100029, China

4, Department of Cardiology, Shanxi Cardiovascular Hospital, Taiyuan, Shanxi, 030001, China

Fanqi Li and Xiaofang Li contributed equally to this article.

Address correspondence to:

Prof. Qiming Liu

The Second Xiangya Hospital of Central South University, Changsha 410000, China.

E-mail: qimingliu@csu.edu.cn

Abstract

Background: Guidelines on coronary intermediate lesions strongly recommend deferred revascularization after detecting a normal fractional flow reserve (FFR). Researches about triglyceride to high-density lipoprotein cholesterol (TG/HDL-C) on cardiovascular diseases has also been well conducted. However, the association of TG/HDL-C and long-term adverse clinical outcomes remains unknown for patients deferred revascularization following FFR.

Methods: This study retrospectively included 374 coronary artery disease (CAD) patients with non-significant coronary lesions diagnosed by coronary angiography (CAG) and FFR. The main outcome measure was the combination of major adverse cardiovascular and cerebrovascular events (MACCEs). All patients were categorized into three subgroups in terms of TG/HDL-C tertiles (T1<0.96, 0.96≤T2<1.58, T3 ≥1.58). Three different Cox regression models were utilized to reveal the association between TG/HDL-C and prevalence of MACCEs.

Results: 47 MACCEs were recorded throughout a median monitoring period of 6.6 years. The Kaplan-Meier survival curves showed a higher MACCEs rate occurred in the higher TG/HDL-C group (5.6% vs. 12.9% vs. 19.4%, log-rank p <0.01). After adjustment, patients in T3 suffered a 2.6-fold risk compared to the T1 group (T3 vs. T1: HR 2.55, 95% CI 1.05-6.21, p=0.038; T2 vs. T1: HR 1.71, 95% CI 0.65-4.49, p=0.075; p for trend=0.001). The restricted cubic spline (RCS) analysis demonstrated that the HR for MACCEs rose as TG/HDL-C increased. Both the receiver operating characteristic (ROC) and time-dependent ROC proved the excellent predictive ability of TG/HDL-C.

Conclusion: The study illustrates that TG/HDL-C correlates with the risk of MACCEs in CAD patients deferred revascularization following FFR. TG/HDL-C could serve as a dependable predictor of cardiovascular events over the long term in this population.

Key words: TG/HDL-C, Fractional flow reserve, Coronary artery disease, Prognosis

Introduction

Determining whether revascularization for coronary intermediate stenosis presents a significant challenge for cardiovascular interventionalists, defined as having a 40% to 70% obstruction as visually estimated [1]. FFR, which is calculated based on the mean distal stenosis pressure and aortic pressure, is strongly advocated for assessing these intermediate lesions for its physiologic function detection ability [1, 2]. FFR > 0.8 indicates that the lesion is non-significant coronary stenosis and deferred revascularization is recommended. With evidence accumulation, other determinants on prognosis emerged, including the plaque vulnerability and progression [3], the interference of microvascular dysfunction on FFR [4], and other non-coronary factors. A recent meta-analysis containing 4275 patients deferred revascularization following a negative FFR concluded that the cardiovascular events of diabetic patients was 2.08 folds greater than non-diabetic [5]. Hence, it is crucial to screen high-risk populations for patients deferred revascularization and administer more comprehensive treatment, even if with a normal FFR, in order to decrease the occurrence of MACCEs.

Dyslipidemia, identified as a conventional risk factor for CAD, includes a range of lipid particle disorders, such as low-density lipoprotein cholesterol (LDL-C), HDL-C, total cholesterol, and TG, with the recent addition of lipoprotein (a) to this list. LDL-C has been the subject of extensive researches and lowering LDL-C level has been confirmed an effective therapeutic option by clinical practice. Recently, enormous studies have proved the relationship between components of lipids and CAD, besides LDL-C. Among these factors, TG/HDL-C is closely linked to cardiovascular metabolic diseases [6]. Nonetheless, the effect of this ratio still unknown among patients deferred revascularization following FFR. Thus, the study intended to explore association of TG/HDL-C with long-term cardiovascular events in this group.

Methods

Study population

This study retrospectively included 1500 consecutive participants who received FFR and CAG for coronary intermediate lesions from February 2013 to October 2021, the same as the previous study population [7]. The study design was approved and performed in Beijing Anzhen Hospital, which is a major referral center dedicated to cardiovascular disease. The exclusion criteria were also similar to our previous work and are illustrated in Figure 1. The main difference in this study is that only FFR>0.8 out of the 1500 consecutive patients were included for analysis. Ultimately, 374 patients participated in this study. This study design was approved by The Second Xiangya Hospital of Central South University and Beijing Anzhen Hospital.

Interventional Procedures

FFR and CAG procedures were performed by skilled operators in strict compliance with protocols. Should the CAG reveal intermediate stenosis, as determined by at least two seasoned interventionalists, an FFR evaluation is scheduled to be proceed, contingent upon patient and family consent. FFR findings informed decisions about whether PCI. Patients with a positive FFR results that indicated PCI but family members refused were excluded. The selection of specific stent size and perioperative drugs was at the discretion of the physician. No major complications occurred during interventional operation in all patients.

Baseline information collection and Definition

Baseline information was acquired from electronic medical records system 13 after approval by the ethics review committee. On the morning after admission, a 13 fasting venous blood sample was collected then examined in Department of Laboratory Medicine. Monitoring information was gathered via electronic medical records system and through phone calls until October 2023. Diabetes mellitus and hypertension were diagnosed following globally recognized standards. Smoking status was identified if the individual had smoked within the six months leading up to the follow-up. FFR represents 18 the ratio of the average pressure in distal stenosis to the aortic pressure at maximal myocardial perfusion.

Primary endpoints

The median follow-up period in this study up to 79 3 months. The primary outcome

measured was the composite of MACCEs, encompassing nonfatal stroke, repeat revascularization, all-cause death, and nonfatal myocardial infarction. Nonfatal stroke was characterized by the rapid onset of clinical symptoms indicating cerebral dysfunction persisting for over 24 hours, supported by neuroimaging evidence [8]. The other three diagnoses were also made according to official guidelines. All MACCEs occurrence events were recorded and compared in the three groups stratified by TG/HDL-C tertiles.

Statistical analysis

Continuous variables were commonly showed as either the mean ± SD or the median (P25, P75), on the basis of their distribution normality. One-way ANOVA was utilized for comparing normally distributed data, while the Kruskal-Wallis test was calculated for non-normally distributed data. Categorical variables were depicted as counts and percentages, with chi-square or Fisher's exact test employed for statistical analysis. Patients were stratified into three subgroups according to TG/HDL-C tertiles. The incidence of MACCEs was recorded using Kaplan-Meier curves, and differences among the sets were calculated with the log-rank test. Three different Cox regression models were constructed to explore the relationship between graded TG/HDL-C tertiles and MACCEs risk. Model 1 adjusted for sex and age; Model 2 adjusted for sex, age, diabetes mellitus, BMI, smoking, and hypertension; Model 3 adjusted for sex, age, diabetes mellitus, BMI, smoking, hypertension, EF, CRP, LDL-C, uric acid, and FFR. An RCS analysis using 4 knots was plotted to explore relationship between TG/HDL-C (as continuous variable) and MACCEs risk. TG/HDL-C and FFR were

incorporated in logistic regression to generate a new "predict" variable, which was intended to plot receiver operating characteristic (ROC) curve. ROC curves were utilized to compare the predictive capacity of different markers, and time-dependent ROC analysis examined the predictive power of TG/HDL-C at different time points. Differences in AUCs compared to TG/HDL-C were assessed using the Delong test. Data analysis was performed with SPSS 26.0, and visualization was done with GraphPad Prism 9.5.0 and R language software (R 4.1.3). For all calculations, a significance level of $P < 0.05$ was employed.

Results

Ultimately, this study enrolled a total of 374 patients, and Figure 1 displays the selection process flow chart. The average age was 58.63 ± 9.05 years, and 252 (67.4%) individuals were men. The mean value of TG/HDL-C was 1.49 ± 1.04, and tertiles were $T1<0.96$, $0.96 \leq T2<1.58$, $T3 \geq 1.58$. Throughout a median follow-up duration up to 79 months, 47 cases of MACCEs were documented, accounting for 12.6%.

Baseline characteristics

The baseline information stratified by the TG/HDL-C tertiles were exhibited in Table 1. Patients in T3 group are more likely to have dyslipidemia, higher BMI, LDL-C, TG, TC, TG/HDL-C, Glu, UA, CRP, and lower HDL-C compared to the low TG/HDL-C group ($P<0.05$).

Association between TG/HDL-C and MACCEs

Kaplan-Meier curves were utilized to depict the cumulative risk of adverse clinical outcomes across the three groups (Fig 2). Over the duration of the follow-up, there were 7, 16, and 24 MACCEs occurred in the T1, T2, and T3 groups, respectively. As the tertiles of TG/HDL-C increased, significant escalation happened in the cumulative risk of MACCEs (log-rank *P*=0.0026).

Three different regression models were constructed to analyze the HR of MACCEs for TG/HDL-C. (Table 2). When fully adjusted, the patients in T2 and T3 group encountered a greater risk of MACCEs compared to those in T1 group (Model 3, T2 vs. T1: HR 1.71, 95% CI 0.65-4.49; T3 vs. T1: HR 2.55, 95% CI 1.05-6.21, *p* for trend=0.001).

To further investigate the possible nonlinear relationship, an RCS curve was conducted (Fig 3). The likelihood of MACCE events will significantly rise when TG/HDL-C over 1.28, and HR escalated as the ratio TG/HDL-C increasing.

ROC and time-dependent ROC were utilized to detect the predictive capacity of the ratio TG/HDL-C. The findings indicated that TD/HDL-C exhibited a larger AUC compared to BMI, LDL-C, TG, and HDL-C (Fig 4). The statistical difference of AUCs and the specific AUC values of each indicator were clearly displayed in Table 3. Time-dependent ROC curves also exhibited satisfactory predictive value at different times (Fig 5). For patients with deferred revascularization, the baseline FFR value also has important predictive value for their long-term prognosis. The combination of TG/HDL-C with FFR exhibited better diagnostic efficacy than either one alone (Fig 6, Table 3).

Discussion

This study revealed long-term prognostic value of TG/HDL-C in CAD patients who deferred revascularization following a normal FFR assessment for the first time. First, the research findings indicated that patients with elevated TG/HDL-C were at a greater risk of cardiac events than those with lower ratio. Second, the detrimental effects of TG/HDL-C will be significant and aggravated as the ratio increase. Third, TG/HDL-C could act as a dependable predictive factor to predict MACCEs in patients deferred revascularization following FFR.

Revascularization in CAD patients should follow strict criteria, including those on optimal drug treatment still experiencing angina, and those diagnosed with severe stenosis through coronary angiography (CAG). Nevertheless, the decision of whether perform revascularization for patients with intermediate stenosis cloud be ambiguous when based exclusively on CAG. Several strategies are used in current practice to assess the functional significance of coronary lesions and further guide coronary interventional therapy [8]. Among them, FFR has emerged as the "gold standard" in detecting functional ischemia, supported by a wealth of accumulated evidence. FFR-guided PCI has been proved to yield greater benefits than angiography-guided [9, 10]. As evidence continues to grow, authoritative guidelines have classified FFR as IA recommendation for intermediate stenosis [11]. FFR > 0.8 indicates that the lesion is non-significant coronary stenosis and deferred revascularization is recommended. However, it has become evident that the outcomes of deferred revascularization may not be uniform across all patient subgroups, as the utilization of physiology-guided

revascularization increases. A recent meta-analysis containing 4275 patients deferred revascularization following negative FFR concluded that the cardiovascular events of diabetic patients was 2.08 folds greater than non-diabetic [5]. A large multinational study demonstrated that sex differences existed in the prognosis among patients with negative FFR and the HR of males for patient-oriented composite outcome (including revascularization, myocardial infarction, and death) was 2.07 [12]. On the other hand, the pathophysiologic mechanisms underlying CAD are also worth considering. First, plaque vulnerability and progression are major contributors to MACCEs, which can't be well detected by FFR [13]. The COMBINE OCT-FFR trial revealed that thin-cap fibroatheroma positive patients suffered a five-fold higher risk of MACE despite this patients with a negative FFR [14]. Second, 43 the evaluation of coronary stenosis severity using FFR cloud be partially confounded by microvascular dysfunction [15]. More importantly, both atherosclerotic plaque vulnerability and microvascular dysfunction are associated with dyslipidemia [16]. This study findings uncovered 6 the association between TG/HDL-C ratio, two important particles of dyslipidemia， and long-term adverse clinical outcomes among CAD patients who have a normal FFR.

1 The influence of TG and HDL-C on cardiovascular disease development remains a subject of heated debate, given the unclear effects and contradictory findings from clinical research. Triglycerides are fat molecules formed from 3 molecules of long chain fatty acids and glycerol. HDL-C is a lipoprotein that transports cholesterol from body tissues to the liver. Adequate research confirms the relationship between TG and cardiovascular disease; however, more importantly, there are conflicting conclusions

about the ability of this intervention to achieve significant clinical benefit in randomized controlled studies [17]. The fibrates and omega-3 fatty acids, two classic types of TG-lowering drugs, exerted variable clinical results [18-20]. Similarly, the same phenomenon occurred in HDL-C [21]. With the birth of PCSK9 and clinical practice, more and more studies are focusing on patients achieving optimal LDL-C levels and finding that residual cardiovascular risk will be increased if these patients have combined dyslipidemia, particularly for low HDL-C concentrations and high TG levels [22]. However, it is evident that the ratio of TG to HDL-C is linked with cardiovascular events. For example, the widely studied and hot metric: atherogenic index of plasm, is calculated from TG and HDL-C. This study findings similarly suggest that TG/HDL-C possesses superior predictive capacity than TG and HDL alone. Ample studies have been conducted concerning the connection between TG/HDL-C ratio and severity of CAD, covering aspects including metabolic syndrome, insulin resistance, and the existence of high-risk coronary plaques [23-25]. Besides, TG/HDL-C also linked to adverse cardiovascular outcomes for CAD patients [26, 27]. As previously mentioned, as the shortcomings of FFR in identifying unstable plaques and vulnerability to other factors, this close connection of TG/HDL-C and CAD may play a greater role in patients with immediate lesion to assist in screening high-risk patients. Similar to these studies, this research proved that higher level of TG/HDL-C increased risk of MACCEs in patients deferred revascularization following FFR.

FFR related indicators, such as iFR, post-PCI FFR, CT-FFR, are strongly associated

with prognosis. The reason why FFR is inferior to TG/HDL-C may be related to the following two points in current study. First, we all know that the cutoff value of FFR is controversial, and even the concept of “gray zone”. Second, this research design only included patients with FFR>0.8. The range of FFR changed from 0-1 to 0.8-1, which may diminish the predict power of FFR. Furthermore, integrating FFR with the TG/HDL-C ratio demonstrated the highest predictive capability, indicating that a predictive model that combines interventional assessment and laboratory results could have a more significant impact on clinical treatments.

Study strengths and limitations

This study has several strengths. First, it enriches prognosis information of the population with deferred revascularization following FFR, which has rarely been studied. Second, the prognostic value of TG/HDL-C on long-term incidence of MACCEs were investigated in CAD patients who deferred revascularization following a normal FFR assessment for the first time. Third, attention should be paid to the management of the levels of lipid components in patients, not only LDL-C. On the other hand, several limitations also exist. First, the study was conducted at a single-center and was retrospective in nature, which may introduce selection bias or potential confounding variables. Second, TG and HDL-C were only assessed upon admission, with no record of their dynamic changes throughout the follow-up period.

Conclusion

The study illustrates that TG/HDL-C correlates with the risk of MACCEs in CAD patients deferred revascularization following FFR. TG/HDL-C could serve as a dependable predictor of long term cardiovascular events in this population.

Acknowledgement

Not applicable.

Authors' contributions

Fanqi Li and Xiaofang Li did statistical analysis and wrote the manuscript draft. Fanqi Li, Qiuyu Li, and Donghui Zhao collected the raw data. Fanqi Li, Jiabao Zhou, and Jiayi Zhu followed up the patients. Qiming Liu and Haixiong Wang made substantial revisions to the content of the manuscript. Qiuzhen Lin, Yong Zhou, and Keke Wu searched the literatures. All authors read and approved this version.

Funding

This study was funded by the National Natural Science Foundation of China (Grant No. 82270337, 82070356), the Hunan Provincial Natural Science Foundation of China (Grant No. 2021JJ30033), and the Key Project of Hunan Provincial Science and Technology Innovation (Grant No. 2020SK1013).

Data Availability

The datasets in this study can be obtained from the corresponding author upon

reasonable request.

Declarations

Ethics approval and consent to participate

This study design was approved by The Second Xiangya Hospital of Central South University and The Beijing Anzhen Hospital.

Consent for publication

Not applicable.

Competing interests

All authors declare that they have no competing interests.

References:

1. Patel MR, Calhoon JH, Dehmer GJ, Grantham JA, Maddox TM, Maron DJ, Smith PK: **ACC/AATS/AHA/ASE/ASNC/SCAI/SCCT/STS 2017 Appropriate Use Criteria for Coronary Revascularization in Patients With Stable Ischemic Heart Disease: A Report of the American College of Cardiology Appropriate Use Criteria Task Force, American Association for Thoracic Surgery, American Heart Association, American Society of Echocardiography, American Society of Nuclear Cardiology, Society for Cardiovascular Angiography and Interventions, Society of Cardiovascular Computed Tomography, and Society of Thoracic Surgeons.** *J Am Coll Cardiol* 2017, **69:**2212-2241.
2. Neumann FJ, Sousa-Uva M, Ahlsson A, Alfonso F, Banning AP, Benedetto U, Byrne RA, Collet JP, Falk V, Head SJ, et al: **2018 ESC/EACTS Guidelines on myocardial revascularization.** *Eur Heart J* 2019, **40:**87-165.
3. Kedhi E, Berta B, Roleder T, Hermanides RS, Fabris E, AJJ IJ, Kauer F, Alfonso F, von Birgelen C, Escaned J, et al: **Thin-cap fibroatheroma predicts clinical events in diabetic patients with normal fractional flow reserve: the COMBINE OCT-FFR trial.** *Eur Heart J* 2021, **42:**4671-4679.
4. Kovarnik T, Hitoshi M, Kral A, Jerabek S, Zemanek D, Kawase Y, Omori H, Tanigaki T, Pudil J, Vodzinska A, et al: **Fractional Flow Reserve Versus Instantaneous Wave-Free Ratio in Assessment of Lesion Hemodynamic Significance and Explanation of their Discrepancies. International, Multicenter and Prospective Trial: The FiGARO Study.** *J Am Heart Assoc* 2022, **11:**e021490.
5. Ekmejian A, Sritharan H, Selvakumar D, Venkateshka V, Allahwala U, Ward M, Bhindi R: **Outcomes of deferred revascularisation following negative fractional flow reserve in diabetic and non-diabetic patients: a meta-analysis.** *Cardiovasc Diabetol* 2023, **22:**22.
6. Yang T, Liu Y, Li L, Zheng Y, Wang Y, Su J, Yang R, Luo M, Yu C: **Correlation between the triglyceride-to-high-density lipoprotein cholesterol ratio and other unconventional lipid parameters with the risk of prediabetes and Type 2 diabetes in patients with coronary heart disease: a RCSCD-TCM study in China.** *Cardiovasc Diabetol* 2022, **21:**93.
7. Li F, Zhao D, Li Q, Lin X, Sun H, Fan Q, De Rosa S: **Uric Acid to High-Density Lipoprotein Cholesterol Ratio is a Novel Marker to Predict Functionally Significant Coronary Artery Stenosis.** *Journal of Interventional Cardiology* 2022, **2022:**1-8.
8. Moscarella E, Gragnano F, Cesaro A, Ielasi A, Diana V, Conte M, Schiavo A, Coletta S, Di Maio D, Fimiani F, Calabrò P: **Coronary Physiology Assessment for the Diagnosis and Treatment of Coronary Artery Disease.** *Cardiology Clinics* 2020, **38:**575-588.
9. Tonino PA, De Bruyne B, Pijls NH, Siebert U, Ikeno F, van' t Veer M, Klauss V, Manoharan G, Engstrøm T, Oldroyd KG, et al: **Fractional flow reserve versus angiography for guiding percutaneous coronary intervention.** *N Engl J Med* 2009, **360:**213-224.
10. Zhang D, Lv S, Song X, Yuan F, Xu F, Zhang M, Yan S, Cao X: **Fractional flow reserve versus angiography for guiding percutaneous coronary intervention: a meta-analysis.** *Heart* 2015, **101:**455-462.
11. Wijns W, Kolh P, Danchin N, Di Mario C, Falk V, Folliguet T, Garg S, Huber K, James S, Knuuti J, et al: **Guidelines on myocardial revascularization.** *Eur Heart J* 2010, **31:**2501-2555.
12. Hoshino M, Hamaya R, Kanaji Y, Kanno Y, Hada M, Yamaguchi M, Sumino Y, Hirano H,

Horie T, Usui E, et al: **Sex Differences in Long-Term Outcomes in Patients With Deferred Revascularization Following Fractional Flow Reserve Assessment: International Collaboration Registry of Comprehensive Physiologic Evaluation.** *Journal of the American Heart Association* 2020, **9**.

13. Nogic J, Prosser H, O'Brien J, Thakur U, Soon K, Proimos G, Brown AJ: **The assessment of intermediate coronary lesions using intracoronary imaging.** *Cardiovascular Diagnosis and Therapy* 2020, **10:**1445-1460.
14. Kedhi E, Berta B, Roleder T, Hermanides RS, Fabris E, Ijsselmuiden AJJ, Kauer F, Alfonso F, von Birgelen C, Escaned J, et al: **Thin-cap fibroatheroma predicts clinical events in diabetic patients with normal fractional flow reserve: the COMBINE OCT–FFR trial.** *European Heart Journal* 2021, **42:**4671-4679.
15. van de Hoef TP, Nolte F, Echavarría-Pinto M, van Lavieren MA, Damman P, Chamuleau SA, Voskuil M, Verberne HJ, Henriques JP, van Eck-Smit BL, et al: **Impact of hyperaemic microvascular resistance on fractional flow reserve measurements in patients with stable coronary artery disease: insights from combined stenosis and microvascular resistance assessment.** *Heart* 2014, **100:**951-959.
16. Arvanitis M, Lowenstein CJ: **Dyslipidemia.** *Annals of Internal Medicine* 2023, **176:**ITC81-ITC96.
17. Malick WA, Waksman O, Do R, Koenig W, Pradhan AD, Stroes ESG, Rosenson RS: **Clinical Trial Design for Triglyceride-Rich Lipoprotein-Lowering Therapies.** *Journal of the American College of Cardiology* 2023, **81:**1646-1658.
18. Rubins HB, Robins SJ, Collins D, Fye CL, Anderson JW, Elam MB, Faas FH, Linares E, Schaefer EJ, Schectman G, et al: **Gemfibrozil for the secondary prevention of coronary heart disease in men with low levels of high-density lipoprotein cholesterol. Veterans Affairs High-Density Lipoprotein Cholesterol Intervention Trial Study Group.** *N Engl J Med* 1999, **341:**410-418.
19. Das Pradhan A, Glynn RJ, Fruchart JC, MacFadyen JG, Zaharris ES, Everett BM, Campbell SE, Oshima R, Amarenco P, Blom DJ, et al: **Triglyceride Lowering with Pemafibrate to Reduce Cardiovascular Risk.** *N Engl J Med* 2022, **387:**1923-1934.
20. Nicholls SJ, Lincoff AM, Garcia M, Bash D, Ballantyne CM, Barter PJ, Davidson MH, Kastelein JJP, Koenig W, McGuire DK, et al: **Effect of High-Dose Omega-3 Fatty Acids vs Corn Oil on Major Adverse Cardiovascular Events in Patients at High Cardiovascular Risk: The STRENGTH Randomized Clinical Trial.** *Jama* 2020, **324:**2268-2280.
21. Endo Y, Fujita M, Ikewaki K: **HDL Functions-Current Status and Future Perspectives.** *Biomolecules* 2023, **13**.
22. Reiner Z: **Managing the residual cardiovascular disease risk associated with HDL-cholesterol and triglycerides in statin-treated patients: a clinical update.** *Nutr Metab Cardiovasc Dis* 2013, **23:**799-807.
23. da Luz PL, Favarato D, Faria-Neto JR, Jr., Lemos P, Chagas AC: **High ratio of triglycerides to HDL-cholesterol predicts extensive coronary disease.** *Clinics (Sao Paulo)* 2008, **63:**427-432.
24. Lelis DF, Calzavara JVS, Santos RD, Sposito AC, Griep RH, Barreto SM, Molina M, Schmidt MI, Duncan BB, Bensenor I, et al: **Reference values for the triglyceride to high-density**

lipoprotein ratio and its association with cardiometabolic diseases in a mixed adult population: The ELSA-Brasil study. *J Clin Lipidol* 2021, **15:**699-711.

25. Miki T, Miyoshi T, Suruga K, Ichikawa K, Otsuka H, Toda H, Yoshida M, Nakamura K, Morita H, Ito H: **Triglyceride to HDL-cholesterol ratio is a predictor of future coronary events: a possible role of high-risk coronary plaques detected by coronary CT angiography.** *European Heart Journal* 2020, **41**.
26. Sultani R, Tong DC, Peverelle M, Lee YS, Baradi A, Wilson AM: **Elevated Triglycerides to High-Density Lipoprotein Cholesterol (TG/HDL-C) Ratio Predicts Long-Term Mortality in High-Risk Patients.** *Heart Lung Circ* 2020, **29:**414-421.
27. Zhan X, Yang M, Zhou R, Wei X, Chen Y, Chen Q: **Triglyceride to high-density lipoprotein cholesterol ratio is associated with increased mortality in older patients on peritoneal dialysis.** *Lipids Health Dis* 2019, **18:**199.

20240322204637968654088735453184

ORIGINALITY REPORT

24%
SIMILARITY INDEX

PRIMARY SOURCES

1	www.science.gov<br>Internet	92 words — 3%
2	www.ncbi.nlm.nih.gov<br>Internet	75 words — 2%
3	Fanqi Li, Qiuzhen Lin, Jiabao Zhou, Jiayi Zhu, Yong Zhou, Keke Wu, Qiuyu Li, Donghui Zhao, Qiming Liu. "A high level of uric acid is associated with long-term adverse cardiovascular outcomes in patients received fractional flow reserve with coronary intermediate stenosis", Nutrition, Metabolism and Cardiovascular Diseases, 2024<br>Crossref	69 words — 2%
4	Yunying Huang, Qiuzhen Lin, Yong Zhou, Jiayi Zhu et al. "Amino acid profile alteration in age-related atrial fibrillation", Journal of Translational Medicine, 2024<br>Crossref	33 words — 1%
5	ltcn.eu<br>Internet	29 words — 1%
6	www.frontiersin.org<br>Internet	29 words — 1%
7	Jiwon Kang, Soon-Sun Kwon, Youngjin Lee. "Clinical nurses' work-life balance prediction due to patient	25 words — 1%

safety incidents using classification and regression tree analysis: a secondary data analysis", BMC Nursing, 2024
Crossref

---

8 academic.oup.com — 25 words — 1%
Internet

---

9 Yingkai Li, Songyuan He, Zheng Wu, Wenzheng Li, Wen Jian, Zichao Cheng, Cong Wang, Yuchen Shi, Jinghua Liu. "The predictive value of the triglyceride—glucose index for cardiovascular events in patients with coronary chronic total occlusion", Cardiovascular Diabetology, 2022 — 21 words — 1%
Crossref

---

10 Yike Dai, Heyong Yin, Chongyang Xu, Hongrui Zhang, Ai Guo, Naicheng Diao. "Association of patellofemoral morphology and alignment with the radiographic severity of patellofemoral osteoarthritis", Journal of Orthopaedic Surgery and Research, 2021 — 17 words — 1%
Crossref

---

11 Liling Wu, Xiaodan Wu, Haofei Hu, Qijun Wan. "Association between triglyceride-to-high-density lipoprotein cholesterol ratio and prediabetes: a cross-sectional study in Chinese non-obese people with a normal range of low-density lipoprotein cholesterol", Journal of Translational Medicine, 2022 — 14 words — < 1%
Crossref

---

12 Lin Ding, Zhuo-Fei Bi, Hang Yuan, Xiao-Hui Zhao, Xue-Dan Guan, He-Rui Yao, Yi-Min Liu. "Sarcomatoid Carcinoma in the Head and Neck: A Population-Based Analysis of Outcome and Survival", The Laryngoscope, 2020 — 14 words — < 1%
Crossref

13 Prakash, Ajay. "A Study of Prevalence of Metabolic Syndrome in Depressive Disorder Patients in a Tertiary Care Hospital", Rajiv Gandhi University of Health Sciences (India), 2023 — 14 words — < 1%
ProQuest

14 Zachary Klaassen, Lael Reinstatler, Martha K. Terris, Willie Underwood III, Kelvin A. Moses. "Beyond biology: the impact of marital status on survival of patients with adrenocortical carcinoma", International braz j urol, 2015 — 14 words — < 1%
Crossref

15 "Biomarkers in Cardiovascular Disease", Springer Nature, 2016 — 13 words — < 1%
Crossref

16 I-Ju Chen, Le-Tien Hsu, Ting-Wei Lin, Jau-Yuan Chen. "Relationship between obesity-related parameters and chronic kidney disease in middle-aged and elderly populations in Taiwan: A community-based study", Frontiers in Nutrition, 2022 — 13 words — < 1%
Crossref

17 Huang Xin, Wang Jianan. "UP-REGULATION OF ENDOGENOUS LEPTIN IMPROVES HUMAN MESENCHYMAL STEM CELL SURVIVAL ABILITY IN VITRO AND THIS CELLS PROTECT FATAL CARDIAL MYOCYTES FROM APOPTOSIS", Heart, 2012 — 12 words — < 1%
Crossref

18 www.oaepublish.com — 11 words — < 1%
Internet

19 Jianfeng Liang, Junfen Fu, Youyun Jiang, Guanping Dong, Xiumin Wang, Wei Wu. "TriGlycerides and high-density lipoprotein cholesterol ratio compared with — 10 words — < 1%

homeostasis model assessment insulin resistance indexes in screening for metabolic syndrome in the chinese obese children: a cross section study", BMC Pediatrics, 2015
Crossref

---

20 Yizhi Hou, Brooke K. Mayer. "Improved disinfection byproduct removal using a polysulfone membrane loaded with powdered activated carbon", Water Environment Research, 2023 — 10 words — < 1%
Crossref

---

21 journals.lww.com — 10 words — < 1%
Internet

---

22 Hassan Mirshafiei, Susan Darroudi, Majid Ghayour-Mobarhan, Habibollah Esmaeili et al. "Altered triglyceride glucose index and fasted serum triglyceride high-density lipoprotein cholesterol ratio predict incidence of cardiovascular disease in the Mashhad cohort study", BioFactors, 2022 — 9 words — < 1%
Crossref

---

23 Yuhan Qin, Yong Qiao, Gaoliang Yan, Dong Wang, Chengchun Tang. "Relationship Between Indices of Insulin Resistance and incident Type 2 Diabetes Mellitus in Chinese Adults", Research Square Platform LLC, 2024 — 9 words — < 1%
Crossref Posted Content

---

24 innovareacademics.in — 9 words — < 1%
Internet

---

25 pubmed.ncbi.nlm.nih.gov — 9 words — < 1%
Internet

---

26 Chun-Li Guan, Hong-Tao Liu, Dong-Hui Chen, Xiao-Qing Quan, Wei-Liang Gao, Xue-Yan Duan. "Is elevated triglyceride/high-density lipoprotein cholesterol ratio — 8 words — < 1%

associated with poor prognosis of coronary heart disease? A meta-analysis of prospective studies", Medicine, 2022
Crossref

27 Corcoran, David, Colin Berry, and Keith Oldroyd. "Current frontiers in the clinical research of coronary physiology", Interventional Cardiology, 2015.
Crossref
8 words — < 1%

28 Empen, K.. "Effect of atorvastatin on lipid parameters, LDL subtype distribution, hemorrheological parameters and adhesion molecule concentrations in patients with hypertriglyceridemia", Nutrition, Metabolism and Cardiovascular Diseases, 200304
Crossref
8 words — < 1%

29 He, S.. "Higher ratio of triglyceride to high-density lipoprotein cholesterol may predispose to diabetes mellitus: 15-year prospective study in a general population", Metabolism, 201201
Crossref
8 words — < 1%

30 John M. Canty. "FAME, ISCHEMIA and Outcomes: Coronary Physiology vs. Plaque Instability and Myocardial Infarction", Trends in Cardiovascular Medicine, 2022
Crossref
8 words — < 1%

31 LeMuge Qi, Aihong Zhang, Yanping Zhang, Zhuo Ren, Chen Zhao, Qian Wang, Kaiming Ren, Jiuxu Bai, Ning Cao. "Association between the Triglyceride to High-Density Lipoprotein Cholesterol Ratio and Mortality in Maintenance Haemodialysis Patients", Research Square Platform LLC, 2022
Crossref Posted Content
8 words — < 1%

32 Li Tian. "The relationship between high density lipoprotein subclass profile and plasma lipids concentrations", Lipids in Health and Disease, 2010
8 words — < 1%

Crossref

33 Long Zhou, Jinzhuang Mai, Ying Li, Min Guo, Yong Wu, Xiangmin Gao, Yangfeng Wu, Xiaoqing Liu, Liancheng Zhao. "Triglyceride to high-density lipoprotein cholesterol ratio and risk of atherosclerotic cardiovascular disease in a Chinese population", Nutrition, Metabolism and Cardiovascular Diseases, 2020
Crossref
8 words — < 1%

34 Mijie Guan, Liling Wu, Yuan Cheng, Dongli Qi, Jia Chen, Haiying Song, Haofei Hu, Qijun Wan. "Defining the threshold: triglyceride to high-density lipoprotein cholesterol (TG/HDL-C) ratio's non-linear impact on tubular atrophy in primary membranous nephropathy", Frontiers in Endocrinology, 2024
Crossref
8 words — < 1%

35 Tong Yang, Yijia Liu, Lin Li, Yanchao Zheng, Yang Wang, Jinyu Su, Rongrong Yang, Mingchi Luo, Chunquan Yu. "Correlation between the triglyceride-to-high-density lipoprotein cholesterol ratio and other unconventional lipid parameters with the risk of prediabetes and Type 2 diabetes in patients with coronary heart disease: a RCSCD-TCM study in China", Cardiovascular Diabetology, 2022
Crossref
8 words — < 1%

36 Yahui Liu, Binbin Zhu, Weicen Zhou, Yao Du, Datun Qi, Chenxu Wang, Qianqian Cheng, You Zhang, Shan Wang, Chuanyu Gao. "Triglyceride–glucose index as a marker of adverse cardiovascular prognosis in patients with coronary heart disease and hypertension", Cardiovascular Diabetology, 2023
Crossref
8 words — < 1%

37 Zhuangsen Chen, Haofei Hu, Miaoling Chen, Xueying Luo, Weili Yao, Qian Liang, Fan Yang,
8 words — < 1%

Xinyu Wang. "Association of Triglyceride to high-density lipoprotein cholesterol ratio and incident of diabetes mellitus: a secondary retrospective analysis based on a Chinese cohort study", Lipids in Health and Disease, 2020
Crossref

38 link.springer.com
Internet
8 words — < 1%

39 mdpi-res.com
Internet
8 words — < 1%

40 translational-medicine.biomedcentral.com
Internet
8 words — < 1%

41 www.wjgnet.com
Internet
8 words — < 1%

42 Byoungjin Park, Dong-Hyuk Jung, Hye Sun Lee, Yong-Jae Lee. "Triglyceride to HDL-Cholesterol Ratio and the Incident Risk of Ischemic Heart Disease Among Koreans Without Diabetes: A Prospective Study Using National Health Insurance Data", Research Square Platform LLC, 2021
Crossref Posted Content
7 words — < 1%

43 Georgiana Pintea Bentea, Brahim Berdaoui, Sophie Samyn, Marielle Morissens, Philippe van de Borne, Jose Castro Rodriguez. "Major Adverse Cardiac Events After Fractional Flow Reserve, Instantaneous Wave-Free Ratio, or Angiography-Guided Revascularization in Atrial Fibrillation: A Retrospective Study", The American Journal of Cardiology, 2024
Crossref
6 words — < 1%

44 Le Wang, Hongliang Cong, JingXia Zhang, YueCheng Hu, Ao Wei, YingYi Zhang, Hua Yang, LiBin Ren, Wei Qi, WenYu Li, ChunWei Liu. "Triglyceride to high-density lipoprotein cholesterol ratio predicts all-cause and
6 words — < 1%

cardiovascular mortality in diabetic patients with coronary artery disease on statin treatment", Research Square, 2021
Crossref Posted Content

45	Wenkai Xia, Xiajuan Yao, Yan Chen, Jie Lin, Volker Vielhauer, Hong Hu. "Elevated TG/HDL-C and Non-HDL-C/HDL-C Ratios Predict Mortality in Peritoneal Dialysis Patients", Research Square, 2020<br>Crossref Posted Content	6 words — < 1%
46	Yuji Koide, Toru Miyoshi, Takahiro Nishihara, Mitsutaka Nakashima, Keishi Ichikawa, Takashi Miki, Kazuhiro Osawa, Hiroshi Ito. "The Association of Triglyceride to High-Density Lipoprotein Cholesterol Ratio with High-Risk Coronary Plaque Characteristics Determined by CT Angiography and Its Risk of Coronary Heart Disease", Journal of Cardiovascular Development and Disease, 2022<br>Crossref	6 words — < 1%
47	Zhiwei Deng, Jiacheng Li, Hui Liu, Tong Luo, Yanjing Yang, Minghui Yang, Xiang Chen. "A light-controlled DNA nanothermometer for temperature sensing in the cellular membrane microenvironment", Biosensors and Bioelectronics, 2022<br>Crossref	6 words — < 1%
48	ousar.lib.okayama-u.ac.jp<br>Internet	6 words — < 1%

EXCLUDE QUOTES	OFF	EXCLUDE SOURCES	OFF
EXCLUDE BIBLIOGRAPHY	ON	EXCLUDE MATCHES	OFF
