# Supplementary figures and images for: Triglyceride to high-density lipoprotein cholesterol ratio associated with long-term adverse clinical outcomes in patients deferred revascularization following fractional flow reserve
Source: Lipids Health Dis. 2024 Apr 2;23:96. doi: 10.1186/s12944-024-02093-1 (PMC10985980; doi:10.1186/s12944-024-02093-1)

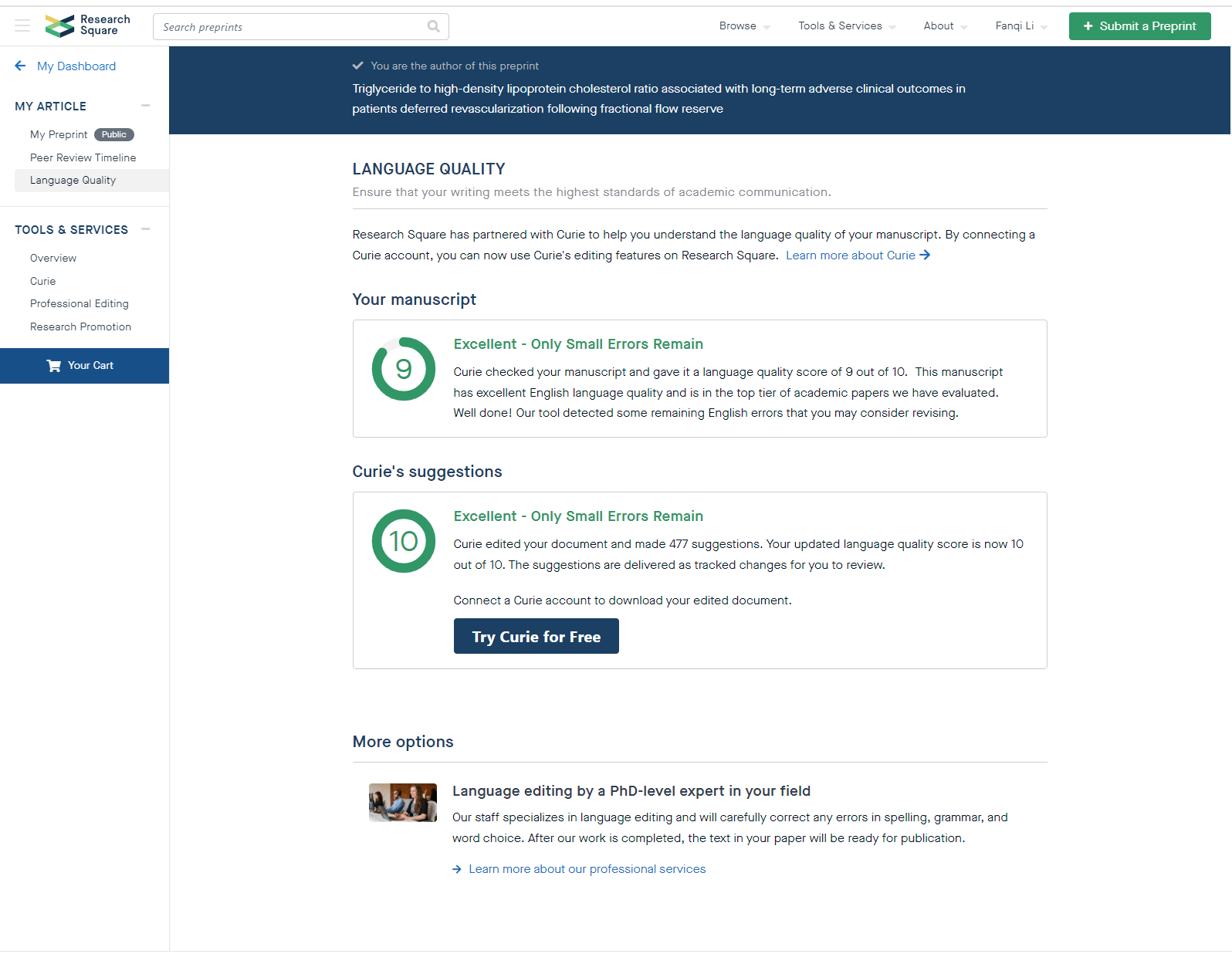

Supplement: Supplementary file 2 — Supplementary Material 2 [file 12944_2024_2093_MOESM2_ESM.png]
